# Clinical practice guideline of the spanish society of oral surgery for oral surgery in patients with coagulation disorders

**DOI:** 10.4317/medoral.26063

**Published:** 2023-06-18

**Authors:** Javier Valenzuela-Mencia, María Ángeles Serrera-Figallo, Daniel Torres-Lagares, Guillermo Machuca-Portillo, Elena Sánchez-Fernández, Eduard Valmaseda-Castellón, María Peñarrocha-Diago, Nuria Fernández-Mosteirín, José Manuel Somoza-Martin, Alba Pérez-Jardón, Cintia Micaela Chamorro-Petronacci, Abel García-García

**Affiliations:** 1 Oral Surgery teaching team. University of Seville; 2 Full Professor in Integrated Dentistry in Special Patients, Oral Medicine and Oral Surgery. University of Seville; 3 Tenured Professor of Oral Surgery. University of Seville; 4 Tenured Professor of Dentistry in Special Patients. Department of Stomatology. Faculty of Dentistry. University of Seville; 5 Full Professor of Oral Surgery. Department of Stomatology. Faculty of Dentistry. University of Granada.; 6 Tenured Professor of Odontostomatology. Faculty of Medicine and Health Sciences. University of Barcelona. Researcher of the group “Dental and Maxillofacial Pathology and Therapeutics” of the Biomedical Research Institute of Bellvitge (IDIBELL); 7 Full Professor of Oral Surgery. Department of Stomatology. Faculty of Medicine and Dentistry. University of Valencia; 8 Medical Area Specialist in Haematology and Haemotherapy. Haemostasis Department. Haematology and Haemotherapy Service. Miguel Servet University Hospital. Zaragoza; 9 Associate Professor. Department of Surgery and Medical-Surgical Specialities. Faculty of Medicine and Dentistry. University of Santiago; 10 Health Research Institute of Santiago de Compostela (IDIS); 11 Health Research Institute of Santiago de Compostela; University of Santiago de Compostela; 12 Tenured Professor of Stomatology. Faculty of Medicine and Dentistry. University of Santiago de Compostela. Health Research Institute of Santiago de Compostela. Head of the Maxillofacial Surgery Service of the University Hospital Clinic of Santiago de Compostela of the Galician Health Service (SERGAS)

## Abstract

**Background:**

The number of patients treated with coagulation disorders, and more specifically with anticoagulant therapy, has increased worldwide in recent years due to increased life expectancy in developed countries. The protocols for managing this type of patient in oral surgery has varied over recent years, especially after the appearance of new direct-acting oral anticoagulants (DOACs). The assessment of risk of bleeding in this type of patient when undergoing a surgical procedure continues to be a controversial issue for patients, dentists and general practitioners. The objective of this document is to offer recommendations, based on evidence, for decision making for patients with coagulopathies who require dental surgical intervention.

**Material and Methods:**

Based on the indications of the “Preparation of Clinical Practice guidelines in the National Health System. Methodological manual”, we gathered a group of experts who agreed on 15 PICO questions based on managing patients with coagulation disorders in dental surgical procedures, such as fitting of implants or dental extractions.

**Results:**

The 15 PICO questions were answered based on the available evidence, being limited in most cases due to the lack of a control group. Two of the PICO questions were answered by the experts with a grade C recommendation, while the rest were answered with grade D.

**Conclusions:**

The results of this review highlight the need to undertake well designed clinical trials with control groups and with a representative sample size.

** Key words:**Oral surgery, coagulopathies, dental extraction, dental implant, oral anticoagulants, heparin, warfarin, acenocoumarol.

## Introduction

The number of patients with coagulation disorders, and with anticoagulant therapy, has increased worldwide in recent years due to increased life expectancy in developed countries. (1). In fact, coumarin derivatives and vitamin K antagonists (VKAs) such as Warfarin (Aldocumar®) and acenocoumarol (Sintrom®) are two of the 100 medications most prescribed in developed countries (2). Although VKAs have been the gold standard anticoagulant treatment for over 60 years, in recent decades, new direct-acting oral anticoagulants (DOACs) have progressively been introduced, whose effects are more predicTable as they have a broader therapeutic range (3).

Management of patients with coagulation disorders, whether acquired or congenital, continues to be an issue that concerns patients, dentists and general practitioners (4,5). Professionals who must decide the guidelines for managing these patients, above all with regard to continuation of anticoagulant/antiaggregant treatment when they will undergo a surgical procedure (6), have doubts due to the protocols having undergone variations in a relatively short period of time (7-9).

Dental extraction is a common procedure, and in the case of anticoagulated patients, dentists usually consult the need to alter the medication guidelines due to the perceived risk of potential uncontrolled bleeding after the dental treatment (10). Shi *et al*. (2017) carried out a meta-analysis which included studies with anticoagulated patients with VKAs and DOACs and ascertained that the risk of postoperative bleeding in anticoagulated patients was greater (RR: 2,7) in comparison with healthy individuals. Nevertheless, the relative risk was significantly greater for patients treated with VKAs (RR=3) than for those treated with DOACs (RR=1.6) (11).

It must be taken into account that current clinical practice guidelines for patients with coagulation disorders are based on studies carried out in different geographic areas of the world, whose idiosyncrasies require a critical view to allow extrapolation of the results globally to our daily practice. The majority of the literature available on the haemorrhagic risk of anticoagulated patients who require a dental extraction and are treated with a coumarin drug revolve around Warfarin (12-15), however, in Spain the coumarin drug used most is acenocoumarol (16).

Another significant aspect is the determination of the ideal INR (International Normalized Ratio) value for treating patients. Some clinical guidelines for managing anticoagulated patients, such as the Scottish Dental Clinical Effectiveness Programme (SDCEP) available on https://www.sdcep.org.uk/media/ypnl2cpz/sdcep-management-of-dental-patients-taking-anticoagulants-or-antiplatelet-drugs-2nd-edition.pdf, recommend not altering the anticoagulant treatment guidelines in patients who have an INR lower than 4, it being recommended that this INR is determined in the 24 hours prior to the dental treatment or with validity of up to 72 hours if its value is usually sTable over time. However, almost half of anticoagulated patients present difficulties achieving adequate control of INR (Spanish Federation of Anticoagulated Patient Associations). In other Spanish guidelines, such as that published by the Spanish Society of Cardiology and the Spanish Society of Epidemiology and Public Health, this value is reduced to an INR of 3, recommending not to treat the patient without consulting the doctor and adjusting the dose of the anticoagulant in case of presenting a greater INR. In line with the main guidelines on cardiology at a global level, it is considered that the desired INR in patients under anticoagulant treatment must be between 2 and 3 (17).

Due to everything described above, this Clinical Practice Guideline (CPG) has been prepared by the Spanish Society of Oral Surgery (SECIB) with the intention of presenting an evaluation of the current evidence on patients with coagulation disorders and helping professionals of health sciences to make clinical decisions.

## Material and Methods

The group of authors of this CPG has followed a rigorous methodological process for its preparation, based on the indications of the document “Preparation of Clinical Practice Guidelines in the National Health System. Methodological manual” of the working group for updating the Manual for the Preparation of Clinical Practice Guidelines in the Spanish National Health System, and the recommendations available on GuíaSalud (https://portal.guiasalud.es/wp-content/uploads/2019/01/manual_gpc_completo.pdf). and the recommendations available on GuíaSalud. Likewise, the scientific bibliography available from December 2011 to December 2021 has been reviewed, following the search strategies included in the CPG (https://portal.guiasalud.es/wp-content/uploads/2023/03/gpc_622_cirugia_bucal_trastornos_coagulacion_secib_compl.pdf). Additionally, for each clinical issue raised, a work sheet was created (own creation) in which the following aspects were detailed: 1) Clinical doubt: Issue on which the authors formulate the clinical question, and which arises from expert knowledge on the subject matter and the professional experience of each of them. 2) PICO question: Question which raises the clinical issue, and which is structured so as to incorporate the Patient (or target population for whom the intervention is intended), the Intervention/Comparison (intervention measured as a comparison or not with another carried out) and Outcome (expected result upon applying the indicated intervention). 3) Introduction: Contextualisation of the scientific knowledge that exists in relation with the specific pathology on the clinical doubt raised, accompanied by the PICO question and the recommendation. 4) Type of question: Classification of the type of question from the following possibilities: epidemiological/etiological, diagnostic, therapeutic or prognostic. 5) Methodology used: This section describes the specific and concrete methodology of the bibliographic search based on: a) searches in databases such as MEDLINE/ PubMed, EMBASE, Cochrane Library; b) the subject heading with the search terms and other Boolean operators (AND, OR, NOT), as well as the use of keywords, Emtree and MeSH; and c) the search results indexing all bibliographic references. 6) Evaluation and synthesis of the evidence: Here the result of the evaluation of each one of the studies referenced in the previous section is listed. The analysis discussed determines the quality of the scientific evidence on which we will base the recommendations made and which it will subsequently be necessary to know to define the “strength” of the recommendation. To carry out the analysis, we use verification templates or checklists of the Scottish Intercollegiate Guidelines Network (SIGN) available on https://www.sign.ac.uk/media/2038/sign50_2019.pdf, to evaluate the randomised clinical trials (RCT), systematic reviews and meta-analysis, cohort studies, case-control studies, studies of diagnostic tests and economic evaluations; OSTEBA datasheets for evaluating case series and the AGREE tools for evaluating the CPGs. 7) Preparation of conclusions: This final point includes the recommendations that the group formulates on the initial PICO clinical question, as well as the grade of this recommendation. The tools that the authors have used to carry out this recommendation (evaluating the quality of the evidence and grading the strength of the recommendation) are called SIGN, from the Oxford Centre for Evidence-Based Medicine (CEBM), the two that have been used being specifically: the Modified SIGN for issues on diagnosis, and the SIGN for the rest of the issues (treatment, prognosis, aetiology, etc.). After cataloguing each piece of evidence found which responds to a PICO question, we give them a degree of recommendation, classified by the letter A, B, C or D and whose meaning can be observed in Table 1.

**Table 1:**
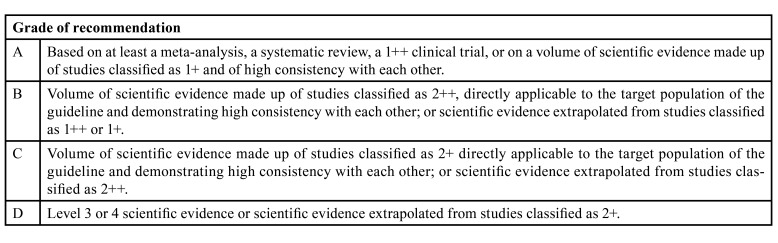
Summary of evidence and recommendations on clinical practice according to GRADE (Grading of Recommendations, Assessment, Development, and Evaluations).

8) Recommendations for future research: In the process of preparing a CPG, knowledge gaps are identified for which the authors may consider it necessary to recommend future lines of research, if they consider them pertinent for patients and professionals. In our case, these proposals are intended to be as specific as possible so that they are useful and understandable for future research projects.

- Development group of the guideline

The SECIB is a scientific association created to offer adequate support to all professionals who call for an up-to-date scientific space for Oral Surgery. To fulfil its objectives, the Association has commissioned a group of experts with developing a CPG on managing patients with coagulation disorders in dental surgical procedures, such as fitting of implants or dental extractions.

This Guideline has been created addressing different cases to cover the widest range of possible scenarios that oral health professionals face with this type of patient. The group that has prepared this CPG is made up of clinical and university experts of the health sciences department: specialists in oral surgery with extensive careers in teaching and research, specialists in the field of haematology and haemotherapy, professionals with extensive experience in managing patients with coagulation disorders in oral surgical procedures, experts in oral surgery, oral medicine and renowned researchers. This CPG has been prepared and evaluated based on the scientific evidence available and is intended to be useful for patients and professionals from different fields: dentists, stomatologists, oral surgeons, maxillofacial surgeons, doctors and clinical specialists of the health sciences department.

- Exhaustive search for scientific evidence

The first approach to the bibliography through the MEDLINE/PubMed database contributed to defining the scope and objectives of the guideline. A first preliminary search of the bibliography was carried out between September and October 2021. The references were downloaded on the Mendeley manager, and thanks to the review by the panel of experts it was possible to detect the important references that had been omitted. The second phase of the search began in October 2021, once the initial approach had been evaluated and it was considered necessary to redefine both the search criteria and the scope of the guidelines themselves. The criteria for developing the questions in PICO format were developed, a task carried out from November 2019 to September 2021, but which continued being completed until December 2021 thanks to weekly alerts, to update the material. The complete texts of these references were shared with the panel of experts through Mendeley. From the exhaustive search of the bibliography, the questions were constructed generating lists of specific issues which could be considered. Criteria for inclusion and exclusion were also defined: in general, any reference which was not written in English, French or Spanish was excluded, and the publication date was limited to the last 10 years. Finally, a selection was carried out on the titles and abstracts of the articles identified in the searches to eliminate those which did not fulfil the search criteria. With regard to sources of information, MEDLINE (through its PubMed interface) and the EMBASE database were used. Search strategies were developed for both with the purpose of restricting or broadening the scope: combination of controlled language vocabulary (the MeSH and Emtree thesauruses) and terms in free language or phrases which appeared in titles or abstracts. The references were stored in a private group on Mendeley, through which there was access to the full text, with a display for marking and making annotations on the references. The articles were pasted into the full text directly on the Mendeley reference manager. Using the Mendeley social module, reviewers were also able to check whether any other article had been published and add their comments. Additionally, for each one of the references, Mendeley directly loaded the associated tags, but added a general tag for identifying to which PICO question or questions that reference corresponded, as well as the type of study (clinical trial, systematic review, meta-analysis, etc.). Finally, a final document was prepared with all the references corresponding to each PICO question of the CPG. The search strategies were reviewed following the document by Sampson *et al*. “An evidence- based practice guideline for the peer review of electronic search strategies” (18).

## Results

1. In anticoagulated patients with a coumarin drug (acenocoumarol or warfarin) who require a dental extraction, is there an increased haemorrhagic risk in comparison with non-anticoagulated patients? And what if they are also taking antiaggregant medication?

Dental extraction in anticoagulated patients with dicoumarinics appears predicTable if the INR ranges are controlled, extracting a single tooth and applying local haemostatic measures to control the haemorrhage. The risk of postoperative bleeding of these patients appears similar to the risk of healthy individuals when the INR value does not exceed 2.2. If we take into account other conditions such as INR greater than 3 or several extractions, the evidence available is limited for answering this question, therefore in these cases it is recommended to contact the patient’s doctor to decide a treatment plan (Grade C recommendation). Without doubt, in this review we have lacked more studies on acenocoumarol, as the majority included patients treated with warfarin or did not specify the vitamin K antagonist medication administered. The studies designed to assess the risk of bleeding must very clearly define aspects such as: the monitoring time of the patient, which we recommend being at least 7 days; the main risk variable, which we recommend being dental extraction, as the medical condition of the patient may vary at different times; the INR range that will be established to include anticoagulated patients; the target population (as the polymorphism of isoenzyme CYP2C9 prevalent in the Asian population affects the clearance of warfarin); as well as also registering whether the patient has previously been treated with antibiotics and concomitant treatment with platelet antiaggregants.

2. In anticoagulated patients with heparin who require a dental extraction, what increase in haemorrhagic risk exists in comparison with non-anticoagulated patients? And what if they are also taking antiaggregant medication?

Evidence on dental extractions in patients treated with heparin mostly refers to anticoagulated patients with acenocoumarol who have been treated with bridge therapy with heparin for a dental extraction. Publications appear to reflect an increased risk of bleeding in comparison with the guideline to continue with the anticoagulant treatment. Nevertheless, it appears that local haemostatic measures should be sufficient for managing to contain the post-extraction haemorrhage in any case (Grade D recommendation). It must be taken into account that the evidence for these results is limited, and it should be expected that in light of reviewed publications, this literature will decrease, as the guideline of not replacing the anticoagulant treatment with subcutaneous heparin appears to have more predicTable results.

3. In anticoagulated patients with DOACs who require a dental extraction, what increase in haemorrhagic risk exists in comparison with non-anticoagulated patients? And what if they are also taking antiaggregant medication?

The evidence on risk of postoperative bleeding after dental extractions in patients treated with DOACs appears to demonstrate an increased risk of bleeding in comparison with healthy individuals (Grade D recommendation). Nevertheless, the evidence for these results is limited and heterogeneous. In the majority of the articles included there is not a significant representation of patients treated with DOACs or of all types commercialised in Spain. The majority of studies are retrospective and apply different protocols in these patients, such as the continuation of the treatment, the interruption of the dose on the day of surgery or suspension up to 5 days before the extraction. This makes controlled clinical trials with a representative sample size of all types of DOACs and healthy individuals necessary.

4. In anticoagulated patients with a coumarin drug (acenocoumarol or warfarin) who require fitting of dental implants, is there an increased haemorrhagic risk in comparison with non-anticoagulated patients? And what if they are also taking antiaggregant medication?

The continuation of the anticoagulant treatment in patients undergoing simple procedures in fitting implants does not entail a significant increase in the risk of haemorrhages compared with stopping or reducing the treatment, therefore its suspension is not justified (Grade D recommendation). Future lines of research should study the potential effect of oral anticoagulants on the risk of haemorrhages through randomised clinical trials with an adequate sample size, evaluating the effect of this type of medication on patients undergoing fitting of implants to allow valid conclusions to be obtained. These trials should be carried out with patients undergoing only implant surgery, as if the patients are undergoing different surgical techniques these results are not conclusive for any of them. Additionally, it should be evaluated whether the joint use of oral anticoagulants and antiaggregant medication alters the haemorrhagic risk, to thereby be able to create protocols which guide clinical practice. Likewise, it should be compared between patients who continue their anticoagulant treatment and those who stop it, to thereby be able to obtain results with sufficient scientific evidence to contribute to guiding clinical practice.

5. In anticoagulated patients with heparin who require fitting of dental implants, what increase in haemorrhagic risk exists in comparison with non-anticoagulated patients? And what if they are also taking antiaggregant medication?

Treatment with heparin in patients undergoing simple procedures in the fitting of implants does not entail a significant increase in the risk of haemorrhages, therefore their continuation during this type of surgery is recommended (Grade D recommendation). The substitution of the oral anticoagulant with heparin in patients undergoing simple procedures in the fitting of implants does not offer benefits with regard to the risk of haemorrhages, therefore this action is not justified. (Grade D recommendation). Future lines of research should study the potential effect of heparin on the risk of haemorrhages through randomised clinical trials, evaluating the effect of this type of medication on patients undergoing fitting of implants to allow valid conclusions to be obtained. These trials should include a large sample of patients, as well as groups where there is a comparison between the continuation of the treatment with heparin, stopping this type of therapy and a control group, to be able to analyse whether these situations alter the risk of haemorrhages. Additionally, it should be evaluated whether the joint use of heparin and antiaggregant medication alters the risk of haemorrhages, to thereby be able to create protocols which guide clinical practice.

6. In anticoagulated patients with a direct-acting oral anticoagulant (DOAC) who require fitting of dental implants, is there an increased haemorrhagic risk in comparison with non-anticoagulated patients? And what if they are also taking antiaggregant medication?

Patients being treated with direct-acting oral anticoagulants may be treated safely with osseointegrated implants. Intraoperative bleeding may be slightly greater, although postoperative bleeding appears to be similar to that of patients who do not take anticoagulants (Grade D recommendation). There is no clinical information for determining the effect that the additional administration of a platelet antiaggregant may have on these patients. Further studies should be carried out including larger samples, as the current samples only have a limited number of cases. It would also be necessary to increase the methodological quality of the studies, as there are numerous biases which limit the strength of the recommendations.

7. In patients with hereditary coagulopathies who require a dental extraction, what increase in haemorrhagic risk exists in comparison with healthy individuals? And what about in patients who require dental implants?

There is not sufficient information on the increased risk of intraoperative or postoperative bleeding in patients with hereditary coagulopathies in comparison with healthy individuals in dental extraction or implant treatments. Dental extractions may be carried out after evaluating the severity of the coagulopathy. In the most serious cases, it is necessary to guarantee a minimum factor before the dental extraction. Cases of haemophilia with inhibitor antibodies must be considered serious and may require greater preparation of the patient. Therefore, there is not sufficient data on the treatment with dental implants in patients with hereditary/congenital coagulopathies, such as haemophilia or Von Willebrand disease. Nevertheless, dental implants may be fitted, as the risk of bleeding appears lower than for dental extractions (Grade D recommendation). Therefore, it must be considered whether it is necessary to carry out general anaesthesia or sedation, avoid trunk anaesthesia techniques, carry out a three-dimensional diagnosis with computed tomography, and take measures to prevent bleeding (local haemostatic measures and evaluating systemic measures such as coagulation factors) before, during and after the intervention (Grade D recommendation). In any case, an individual evaluation of the case should be carried out with the haematology unit or haemophilia centre. Studies with a larger amount of evidence should be carried out, because to date only case series, isolated clinical cases, documents by experts and a clinical practice guideline are available, and therefore there are no studies with grade 2 evidence. Specific studies should be carried out to select larger samples of patients with each coagulopathy, especially the most frequent, such as haemophilia A and Von Willebrand disease, in order to prepare protocols or specific guidelines for each one. The methodological quality of the studies should also be increased, designing observational studies that may offer a minimum of grade 2 evidence.

8. In anticoagulated patients with a coumarin drug (acenocoumarol or warfarin) who require a dental extraction, what reduction of haemorrhagic risk is produced by local haemostatic measures in comparison with non-anticoagulated patients? And what if they are also taking antiaggregant medication?

In anticoagulated patients undergoing simple extractions it is not recommended to stop the anticoagulant treatment, recommending the application of local haemostatic measures (Grade C recommendation). No local haemostatic measure has been demonstrated as superior to the others, therefore it is recommended to use those which have more evidence, such as sutures, collagen or cellulose sponges, as well as compression with gauze soaked in tranexamic acid (Grade C recommendation). The appearance of postoperative haemorrhages is associated with the age of the patient, therefore in older patients undergoing simple extractions the use of local haemostatic measures is recommended (Grade C recommendation). Future lines of research should study the effect of different local haemostatic measures through randomised clinical trials evaluating the effect of these measures on anticoagulated patients undergoing extractions to allow valid conclusions to be obtained. These trials must be carried out on large samples of patients where the anticoagulation is not stopped and with INR in the range recommended in the main guidelines and protocols. With regard to the above, patients with an INR of between 2 and 4 should be included, as it is common to find patients with INR levels over 3, and to thereby be able to evaluate whether these haemostatic measures continue to be effective for such INR values. Additionally, the effectiveness of these haemostatic measures should be studied in patients who combine anticoagulant and antiaggregant treatment, to thereby be able to create protocols which guide clinical practice in this type of patient.

9. In anticoagulated patients with heparin who require a dental extraction, what reduction of haemorrhagic risk is produced by local haemostatic measures in comparison with non-anticoagulated patients? And what if they are also taking antiaggregant medication?

Due to the current lack of evidence, future lines of research should focus on being able to answer this PICO question through well designed clinical trials evaluating the effectiveness of the different local haemostatic measures in patients treated with heparin undergoing extractions. In anticoagulated patients with heparin and undergoing extractions, it is recommended to carry out an exhaustive anamnesis of the patient to ascertain the time they have been treated and to precisely know the half-life of the heparin used. The surgical procedure should be carried out at the time of maximum decrease of the plasma concentrations of the drug, to then continue with the anticoagulant treatment, simultaneously applying local haemostatic measures, such as compression with dry gauze or preferably soaked in tranexamic acid, the suture of the wound or the use of different types of haemostatic sponges or dressings (Grade D recommendation).

10. In anticoagulated patients with DOACs who require a dental extraction, what reduction of haemorrhagic risk is produced by the local measures in comparison with non-anticoagulated patients? And what if they are also taking antiaggregant medication?

Due to the lack of quality scientific evidence in the literature on this issue, an expert opinion is proposed. In anticoagulated patients with a direct anticoagulant drug and undergoing extractions, it is recommended to precisely know the half-life of the drug used, the dental extraction having to be carried out at the time of maximum decrease of the plasma concentrations of the specific anticoagulant, delaying its administration at least 4 hours after the extraction, simultaneously applying local haemostatic measures such as sutures, different haemostatic sponges or dressings, or compression with dry gauze or preferably soaked in tranexamic acid (Grade D Recommendation). Taking into account the current limited evidence and its methodology, future lines of research should focus on being able to answer this PICO question through well designed clinical trials evaluating the effectiveness of the different local haemostatic measures in patients treated with direct anticoagulants undergoing extractions compared with healthy controls and with an adequate sample size.

11. In anticoagulated patients with a coumarin drug (acenocoumarol or warfarin) who require a dental extraction, what reduction of haemorrhagic risk is produced the PHARMACOLOGICAL TREATMENT in comparison with non-anticoagulated patients? And what if they are also taking antiaggregant medication?

Due to the lack of quality scientific evidence in the literature on this issue, an expert opinion is proposed. In anticoagulated patients with a coumarin drug and undergoing extractions the continuation of the oral anticoagulant treatment is recommended, with concomitant use of local haemostatic measures consisting of sutures, compression with dry gauze or preferably soaked in tranexamic acid, haemostatic sponges or dressings, without the need to use pharmacological treatment to reduce the haemorrhagic risk (Grade D recommendation). Future lines of research should focus on being able to answer this PICO question through well designed clinical trials evaluating the effectiveness of the different pharmacological treatments aimed at promoting haemostasis in anticoagulated patients with coumarin drugs undergoing extractions.

12. In anticoagulated patients with heparin who require a dental extraction, what reduction of haemorrhagic risk is produced by the PHARMACOLOGICAL TREATMENT in comparison with non-anticoagulated patients? And what if they are also taking antiaggregant medication?

In anticoagulated patients with heparin and undergoing extractions it is recommended to use local haemostatic measures consisting of sutures, compression with dry gauze or preferably soaked in tranexamic acid, haemostatic sponges or dressings, without the need to use pharmacological treatment to reduce the haemorrhagic risk (Grade D recommendation). Future lines of research should focus on being able to answer this PICO question through well designed clinical trials evaluating the effectiveness of the different pharmacological treatments aimed at promoting haemostasis in patients treated with heparin undergoing extractions.

14. In patients with hereditary coagulopathies who require a dental extraction, what reduction in haemorrhagic risk is produced by local haemostatic measures in comparison with healthy individuals? And what about in patients who require dental implants?

When making a recommendation on this issue, there is no article that has fully addressed the PICO question that we raise, as none have studied the reduction of haemorrhagic risk in comparison with healthy individuals. The level of recommendation of this section is the opinion of the experts signing the guideline (Grade D recommendation). With regard to purely mechanical measures, we have not found bibliography where they are applied only for the haemorrhagic control, as today they are usually used with obligatory consultation of the haematologist and combined with pharmacological measures. There are hardly any studies in this group of patients for whom dental implants are fitted, therefore the evidence is insufficient. Among the most important considerations are: the preparation of a treatment plan after preparing the clinical history and consulting the haematologist, the treatment being carried out first thing in the morning and at the start of the week, and undertaking the dental procedures in a single session whenever possible. Future lines of research should focus on being able to answer this PICO question through well designed clinical trials evaluating the effectiveness of local measures aimed at promoting haemostasis in patients with congenital coagulopathies undergoing extractions.

15. In patients with hereditary coagulopathies who require a dental extraction, what reduction in haemorrhagic risk is produced by PHARMACOLOGICAL TREATMENT in comparison with healthy individuals? And what about in patients who require dental implants?

When making a recommendation on this issue, no article has fully studied the PICO question that we raise. There is no conclusive evidence, and through the bibliography we cannot answer this question. The level of recommendation of this section is the opinion of the experts signing the guideline (Grade D recommendation). Based on our clinical experience, the pharmacological treatment must always be carried out through consultation with the haematologist. Although sufficient, there is more literature on antifibrinolytic treatment in dental extractions than in implants. In case of doubt, the surgery must be carried out in a hospital environment. The authors of the articles included in this review highlight that further research is necessary to improve the base of evidence in treatment of people with haemophilia and Von Willebrand disease, as well as other rare congenital disorders.

External validation

The methodological support team was made up of a technical expert on methodology and healthcare based on evidence, and a librarian specialised in the field of Health. Additionally, an external methodological support model was used (19), and for reasons of effectiveness and efficiency, an external consultant was incorporated in the preparation and development of projects (20).

## Discussion

The number of patients treated with coagulation disorders and, more specifically, with anticoagulant therapy has increased throughout the world in recent years due to the increase in life expectancy in developed countries.

Protocols for the treatment of this type of patients in oral surgeries have changed in recent years, especially after the appearance of the new direct oral anticoagulants (DOACs). Evaluation of the risk of bleeding in this type of patient when undergoing a surgical procedure continues to be a controversial issue for patients, dentists, and family physicians.

The professionals who must decide the guidelines for the treatment of these patients, especially with regard to the continuation of anticoagulant/antiplatelet treatment when they are going to undergo a surgical procedure, have doubts because the protocols have undergone variations over a relatively short period of time.

This guide is aimed at professionals in Dentistry and Stomatology, whether they are generalists or specialists, who practise oral surgery in university centres, dental clinics, primary care centres or hospitals, both in the public and private spheres. Consultation of these recommendations, based on current scientific evidence, should help make therapeutic decisions that promote excellence in oral surgery in patients with coagulation disorders.

This guide intervenes in different areas of the surgical care process in patients with coagulation disorders. It allows the user to know the relevant aspects to consider in patients with coagulopathies, as well as the available evidence to proceed with them. It also makes professionals aware of the importance of individualising the cases of anticoagulated patients and the value of interconsultations between health professionals.
